# The Regulatory Role of EvfG Through Coordinated Control of Flagellar Biosynthesis and Energy Metabolism in Porcine Extraintestinal Pathogenic *Escherichia coli* (ExPEC)

**DOI:** 10.3390/biology14070822

**Published:** 2025-07-07

**Authors:** Bingbing Zong, Peiyi Wang, Wei Liu, Aihua Wu, Yong Xiao, Shulin Fu, Yinsheng Qiu, Yanyan Zhang, Wentong Liu

**Affiliations:** 1 Hubei Key Laboratory of Animal Nutrition and Feed Science, Wuhan Polytechnic University, Wuhan 430023, China; zongbingbing@whpu.edu.cn (B.Z.); 20220411015@whpu.edu.cn (P.W.); 20230411021@whpu.edu.cn (W.L.); 20240411012@whpu.edu.cn (A.W.); 924518497@webmail.hzau.edu.cn (Y.X.); shulinfu@whpu.edu.cn (S.F.); qiuyinsheng6405@whpu.edu.cn (Y.Q.); 2 Engineering Research Center of Feed Protein Resources on Agricultural By-Products, Ministry of Education, Wuhan Polytechnic University, Wuhan 400023, China; 3 Hubei Collaborative Innovation Center for Animal Nutrition and Feed Safety, Wuhan 400023, China; 4 School of Basic Medicine, Hubei University of Arts and Science, Xiangyang 441000, China

**Keywords:** porcine ExPEC, EvfG, motility, energy metabolism, flagellar assembly

## Abstract

In this study, we found that the deletion of the gene *evfG* in the type VI secretion system (T6SS) gene cluster significantly affected the motility of porcine extraintestinal pathogenic *Escherichia coli* (ExPEC) strain PCN033. Furthermore, the bacterial motility assay showed that Δ*evfG* mutants exhibited reduced motility compared to the parental strain. Transmission electron microscopy (TEM) showed a significant reduction in the number of flagella in the mutant Δ*evfG* when compared with PCN033. To further explore the reasons why the deletion of *evfG* affects the motility of PCN033, transcriptomic and metabolomic analyses were conducted. The omics analyses showed that 134 differentially accumulated metabolites and 2236 differentially expressed genes were identified between the mutant Δ*evfG* and the parental strain PCN033. The metabolome profile and functional annotation analyses indicated that the impaired motility of Δ*evfG* was connected to the downregulation of the expression levels of genes associated with the energy metabolism pathway and flagellar assembly. Our study provides a new insight into the diminished PCN033 motility induced by *evfG* deletion. Moreover, the candidate genes and metabolites regulated by the gene *evfG* in the T6SS, which was involved in the motility of PCN033, were reported in this study.

## Simple Summary

Extraintestinal pathogenic Escherichia coli (ExPEC) is a pathogen that can invade and colonize multiple tissues outside the intestine, leading to diseases such as sepsis, immune damage and meningitis. In recent years, the isolation rate of porcine ExPEC has been increasing and exhibits multidrug resistance, which poses severe challenges to the prevention and control of porcine ExPEC. The correct assembly and drive of flagellar-associated gene expression are essential for the motility and pathogenicity of ExPEC. This study was conducted to elucidate the regulator role of EvfG in porcine ExPEC PCN033. We found that the deletion of the gene *evfG* in the type VI secretion system gene cluster significantly affected the motility of porcine ExPEC PCN033. The metabolome profile and functional annotation analysis indicated that the motility of Δ*evfG* was inhibited by the energy metabolism pathway and the downregulation of gene expression levels related to flagellar assembly. This research confirmed that *evfG* can affect the motility of PCN033 by regulating energy metabolism and the expression of flagellar genes, while also providing a theoretical basis for strategies aimed at preventing and controlling infections caused by porcine ExPEC PCN033.

## 1. Introduction

Extraintestinal pathogenic *Escherichia coli* (ExPEC) is a pathogen that can invade and colonize multiple tissues outside the intestine, leading to diseases such as sepsis, immune damage and meningitis [1,2,3]. The isolation rate of porcine ExPEC has been rising in recent years due to China’s swine breeding industry’s rapid development. Additionally, the majority of isolated strains displayed multidrug resistance, which presents significant obstacles to ExPEC prevention and management [4,5,6]. In addition, numerous studies have reported on the similarities between porcine ExPEC and human ExPEC in terms of serogroup and pathogenic characteristics, suggesting that porcine ExPEC may be a zoonotic pathogen and could pose a threat to public health [7,8,9]. At present, there are no effective preventive and therapeutic measures for porcine ExPEC; therefore, the pathogenic mechanism of porcine ExPEC requires further study [10].

The type VI secretion system (T6SS), recognized as a multiprotein apparatus, is widely found in Gram-negative bacteria. It facilitates the delivery of toxins, proteins, and various molecules from bacterial cells into their target cells [11,12,13]. The T6SS gene cluster is usually composed of 15–25 genes, including *hcp*, *vgrG*, *clpV*, *icmF* and so on, in the bacterial genome [14,15,16]. Thus, T6SS is assembled by complex components to form a needle-like hollow tube structure. During the process of bacterial pathogenicity, the T6SS assists bacteria to invade and infect host cells, leading to the development of the disease [17,18]. In addition, T6SS is involved in bacterial collective behavior, biofilm formation, and antibiotic resistance [19,20]. Some reports have shown that when the core gene or effectors of T6SS are missing, the competitiveness and pathogenicity of ExPEC are significantly decreased, highlighting the crucial role of T6SS in the survival and pathogenesis of porcine ExPEC [21,22,23]. Previous studies have reported that EvfG in the T6SS in the human ExPEC RS218 strain is a multifunctional protein that can inhibit bacterial flagellar synthesis, affect bacterial motility, and is also related to bacterial virulence, oxidative stress, and drug transport [16]. However, in porcine ExPEC, previous research has only reported that the EvfG encoded by gene *evfG* in the T6SS gene cluster is a nonclassical protein of unclear function [14,15]. The function of the gene *evfG* in the T6SS gene cluster on ExPEC pathogenicity is still unclear. Although we found that the deletion of gene *evfG* significantly diminished the motility of PCN033 and led to a noticeable decrease in the flagella count of PCN033 in this investigation, the relevant mechanism has not been reported yet. Therefore, this study aimed to reveal the mechanism by which gene *evfG* regulates flagellar assembly to affect porcine ExPEC movement and subsequently impact pathogenicity.

The movement of bacteria is crucial during the initial phases of pathogenic bacterial infections, as well as in the processes of colonization and biofilm development [24]. Depending on the growth environment, *Escherichia coli* (*E. coli*) has two common states of movement: moving independently in large amounts of liquid (swimming); and moving with other cells in a liquid film on a wet surface (swarming). The thrust of both modes of motion is generated by the rotating spiral flagella [25]. The movement of the flagella not only helps the bacteria to better adapt to the environment, but is also closely related to its chemotaxis, adhesion, virulence, and pathogenicity. The flagellum’s migration to host cells is crucial for colonization and is required for adhesion and invasion [26]. Flagella also facilitate the escape of bacteria from infected macrophages and return to the broader environment of the host [27]. Therefore, it is of great significance, for revealing porcine ExPEC pathogenic mechanisms, to study the mechanism by which gene *evfG* affects porcine ExPEC movement.

Recently, transcriptomics and metabolomics have been widely used to study the survival and pathogenic mechanisms of pathogenic bacteria [28,29]. To elucidate the mechanism by which T6SS EvfG regulates the motility of porcine ExPEC PCN033, liquid chromatography-mass spectrometry (LC-MS) was employed to identify differential accumulation metabolites (DAMs) in PCN033 (parental strain) and Δ*evfG* (mutant). Additionally, RNA-Seq was used to analyze differentially expressed genes (DEGs), which was later confirmed through qRT-PCR. This research clarified the genes and metabolites impacted by the *evfG* gene concerning the motility of ExPEC PCN033, while also providing a theoretical basis for strategies aimed at preventing and controlling infections caused by porcine ExPEC PCN033.

## 2. Materials and Methods

### 2.1. Bacteria and Culture Conditions

The porcine ExPEC strains (parental strain PCN033, mutant Δ*evfG* of PCN033 and its complementary strain Δ*evfG*-EvfG) used in this study were donated by the State Key Laboratory of Agricultural Microbiology and stored at our lab. The porcine ExPEC strain PCN033 was isolated from the brain tissue of diseased pigs at a pig farm in Hunan Province (China) in 2006; its serotypes and virulence genes were analyzed in 2012 [4]. All strains were cultured in either Luria-Bertani (LB) broth or on LB agar plates.

### 2.2. Bacteria Motility Assays

The motility assay was conducted using a method reported previously with slight modifications [30,31,32]. For the analysis of swimming motility, PCN033, Δ*evfG*, and Δ*evfG*-EvfG were cultured on semi-solid agar (comprising 1% tryptone, 0.5% sodium chloride, and 0.3% agar) at 30 °C for 14 h. Furthermore, for the assessment of swarming motility, these strains (PCN033, Δ*evfG*, and Δ*evfG*-EvfG) were cultivated on solid agar (containing 1% tryptone, 0.5% sodium chloride, 0.4% agar, and 0.3% beef extract) at 37 °C for 12 h. By comparing the motility ability of the mutant with the parental strain and the motility ability of the complementary strain with the mutant strain, the impact of *evfG* deletion on the motility (both swimming and swarming) of PCN033 was elucidated.

### 2.3. Bacterial Flagella Visualization with Transmission Electron Microscopy (TEM)

TEM was conducted, as outlined in previous studies [32,33]. Initially, the bacterial strains—including the parental strain PCN033, the mutant strain Δ*evfG*, and its complementary strain Δ*evfG*-EvfG—were grown overnight in LB media. Following this incubation, these cultures were diluted 1:1000 into new media and permitted to grow at 37 °C until they achieved an optical density (OD_600_) of 0.6 to 0.8. After centrifuging the bacterial cells for 20 min at a speed of 1000 rpm, they were rinsed with phosphate-buffered saline (PBS). After that, aliquots of the suspension cells were placed onto copper grids that featured a mesh size of 200–400, negatively stained for one minute with 1% phosphotungstic acid, and then photographed using a TEM.

### 2.4. Total RNA Extraction, Library Building, and Transcriptome Sequencing

Total RNA was obtained from PCN033 (*n* = 3) and Δ*evfG* (*n* = 3) utilizing the TRIzol reagent (Takara, Dalian, China). To eliminate genomic DNA from the extracted total RNA, DNase I (Takara) was applied. An RNA-seq transcriptome library was constructed from 2 µg of RNA employing the TruSeqTM RNA Sample Preparation Kit (Illumina, San Diego, CA, USA). Initially, ribosomal RNA (rRNA) was removed with the Ribo-Zero Magnetic Kit (epicenter). Subsequently, a fragmentation buffer was employed to cleave the mRNA into smaller fragments (approximately 200 bp). The fragmented mRNA was reverse transcribed into cDNA through qRT-PCR with the SuperScript Double-Stranded cDNA Synthesis Kit (Invitrogen, Carlsbad, CA, USA) and random hexamer primers (Illumina). During the synthesis of the second strand, dTTP was substituted with dUTP. After the synthesis, the cDNA underwent end-repair and phosphorylation processes. Subsequently, an “A” base was added to the cDNA following Illumina’s protocol. The second-strand cDNA containing dUTP was then identified and broken down by the UNG enzyme. The library was made using 200 bp cDNA target fragments and 2% low-range ultra-agarose. These fragments were measured with TBS380 following 15 PCR cycles of amplification using Phusion DNA polymerase. Finally, a paired-end RNA-seq sequencing library was developed using an Illumina Novaseq with a read length of 2 × 150 bp.

### 2.5. Transcriptome Data Analysis

Base calling was used to transform the raw data from Illumina Hiseq sequencing and the output was saved in FASTQ file format. The reference sequences (GenBank: CP006632.1) were mapped to the assembled sequences and individual genes using Bowtie with default parameters. The abundance of genes and transcripts was assessed through the use of FPKM (fragments per kilobase million). The transcriptome data were analyzed on Majorbio’s (Shanghai, China) cloud platform. Furthermore, the DEGs between PCN033 and Δ*evfG* were identified using DESeq2, with *p*-values adjusted for multiple testing via Benjamini/Hochberg (BH). DEGs were defined as having adjusted *p* < 0.05 and fold change ≥2 or ≤1. KEGG analysis was performed using an R script; GO analysis of DEGs was conducted via Goatools.

### 2.6. Metabolite Extraction

After growing in LB media overnight, the strain PCN033 and Δ*evfG* were transferred at a 1:1000 ratio into a new medium and allowed to develop to an OD_600_ of 0.6–0.8 at 37 °C. After centrifuging the bacterial cells for five minutes at 12,000 rpm, they were rinsed with phosphate-buffered saline (PBS). After precisely weighing a 50 mg solid sample, the metabolites were extracted using an internal standard of 0.02 mg/mL L-2-chlorophenylalanin and a 400 µL methanol:water (4:1, *v*/*v*) solution. After being instantly pulverized for 6 min at −10 °C and 50 Hz in a high-throughput tissue crusher (Wonbio-96c, Shanghai Wanbo Biotechnology Co., Ltd., Shanghai, China), the mixture was treated for 30 min at 5 °C and 40 kHz in an ultrasonic cleaner (SBL-10TD, Ningbo Xinzhi Biotechnology Co., Ltd., Ningbo, China). After that, the samples were kept for 30 min at −20 °C. Following centrifugation at 13,000× *g* for 15 min at 4 °C, the supernatant was meticulously collected and placed into sample vials for analysis using LC-MS.

### 2.7. Metabolite Extract LC-MS and Quality Control (QC) Measurements

An LC-MS system was used to investigate metabolite extracts. The system consisted of a Q-Exactive mass spectrometer (Thermo Scientific™, Waltham, MA, USA) coupled with a Vanquish Horizon UHPLC system (Thermo Scientific™). An HSS T3 column (100 mm × 2.1 mm i.d., 1.8 μm particle size, Waters, Milford, MA, USA) was filled with 2 μL of the extracts. The mobile phases were composed of solvent A (0.1% formic acid in water:acetonitrile, 95:5, *v*/*v*) and solvent B (0.1% formic acid in acetonitrile:isopropanol:water, 47.5:47.5:5, *v*/*v*). The gradient elution program was as follows: 0–0.1 min, 0% B increasing to 5% B; 0.1–2 min, 5% B increasing to 25% B; 2–9 min, 25% B increasing to 100% B; 9–13 min, maintaining 100% B; 13–13.1 min, decreasing from 100% B to 0% B; 13.1–16 min, holding at 0% B. The injection volume was set to 2 µL. The flow rate was maintained at 0.4 mL/min under a temperature of 40 °C. Data were obtained through continuous scanning in both positive and negative ionization modes. The electrospray ionization (ESI) source parameters were configured as follows: mass-to-charge (*m*/*z*), 70–1050; sheath gas flow rate of 40 arbitrary units (arb); auxiliary gas flow rate of 10 arb; auxiliary gas heater temperature of 400 °C; capillary temperature of 320 °C; ESI+ spray voltage of +3500 V; ESI− spray voltage of −2800 V; and normalized collision energy levels of 20, 40, and 60 V. The mass spectrometer operated at a resolution of 70,000 for full-scan MS and 17,500 for tandem MS/MS experiments. Data collection was carried out in Data Dependent Acquisition (DDA) mode.

### 2.8. Metabolite Data Analysis

Metabolite data were analyzed with the help of Progenesis QI (Waters Corporation, Milford, CT, USA) to generate a 3D CSV matrix, which included details about the samples, the names of metabolites, and the intensity of mass spectral responses. After removing internal standard peaks, noise, column bleed, and derivatization artifacts, data were deduplicated and peak-pooled. Metabolic features were identified via precise mass analysis, MS/MS fragmentation, and isotope ratio matching against METLIN and HMDB.

These data were uploaded to the Majorbio cloud platform (https://cloud.majorbio.com, accessed on 26 June 2025) for further database-driven analysis. At least 80% of the identified metabolic characteristics were preserved in each batch of samples. Each metabolic feature was standardized by sum, and values below the lower limit of quantification in specific samples were imputed using minimal metabolite levels. Sum normalization was applied to the sample mass spectral peak intensities to generate the normalized data matrix. After eliminating the QC samples with variables exhibiting a relative standard deviation exceeding 30%, the resulting data matrix was subjected to a logarithmic transformation (log10) in preparation for further analysis.

The data array that had undergone preprocessing was subsequently analyzed using principal component analysis (PCA) and orthogonal partial least squares discriminant analysis (OPLS-DA) through the R package (Version 1.6.2). Model stability was evaluated using seven-fold cross-validation. DAMs were identified based on VIP values from the OPLS-DA model combined with Student’s t-tests, with significance thresholds set at VIP > 1 and *p* < 0.05. In order to determine the associations of differential metabolites with specific pathways, metabolic pathway annotation was performed utilizing the KEGG database. The pathway enrichment analysis was carried out using the Python packages scipy.stats (Version: 1.16.0, https://docs.scipy.org/doc/scipy/, accessed on 26 June 2025), while Fisher’s exact test was employed to identify the biological pathways most closely associated with the experimental treatments.

### 2.9. Bacterial RNA Extraction and qRT-PCR Analysis

Parental strain PCN033 and mutant Δ*evfG* were cultured overnight in LB medium, then subcultured into a fresh medium at a 1:1000 dilution and incubated at 37 °C until an OD_600_ of 0.6–0.8 was reached. Then, bacterial cells were collected by centrifugation at 12,000 rpm for 5 min. The transcriptional level of PCN033 and mutant Δ*evfG* was assessed using reverse transcription-quantitative polymerase chain reaction (qRT-PCR). As directed by the manufacturer, the Bacteria RNA Extraction Kit (Vazyme, Nanjing, China) was used to extract the total RNA. Next, using ABScript Neo RT Master Mix for qPCR with gDNA Remover (ABclonal, Wuhan, China), 1 μg of total RNA was reverse-transcribed. The Bright Cycle Universal SYBR Green qPCR Mix with UDG (ABclonal, Wuhan, China) was used in the qRT-PCR reactions, which were carried out using the QuantStudioTM 1 Plus Real-Time PCR System (Thermo Fisher, Shanghai, China). Results were normalized relative to *E. coli* 16S rRNA and determined using the 2^−ΔΔCT^ method. A comprehensive list of all primers utilized for RT-PCR can be found in Table 1.

### 2.10. Statistical Analysis

The analysis of the study’s data was conducted using GraphPad Prism 8 and IBM SPSS Statistics 23. For comparisons between two groups, unpaired two-sided t-tests were employed, while one-way ANOVA was utilized for comparisons involving more than three groups. Significance levels were indicated as follows: # *p* < 0.05, ## *p* < 0.01, ### *p* < 0.001; * *p* < 0.05, ** *p* < 0.01, *** *p* < 0.001.

## 3. Results

### 3.1. The Deletion of Gene evfG Affects Bacterial Swimming and Swarming

**Figure 1:**
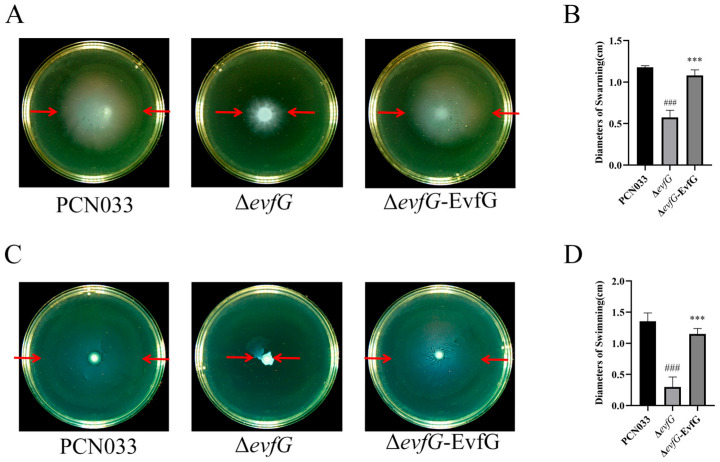
Motility comparison of parental strain, mutant Δ*evfG*, and the complementary strain Δ*evfG*-EvfG: (**A**) swarming abilities of PCN033, Δ*evfG*, and Δ*evfG*-EvfG on a solid-agar medium. The area indicated by the red arrow represents the outer margin of the bacterial halo; (**B**) analysis of the diameter of bacterial swarming in Figure 1A (one-way ANOVA was used to determine statistical significance in comparison to the PCN033 group. ### *p* < 0.001. The unpaired Student’s two-sided t-test was used to evaluate the statistical significance between Δ*evfG* and Δ*evfG*-EvfG. *** *p* < 0.001); (**C**) swimming abilities of these strains on semi-solid agar; and (**D**) analysis of the diameter of bacterial swimming in Figure 1C (one-way ANOVA was used to determine statistical significance in comparison to the PCN033 group. ### *p* < 0.001. The unpaired Student’s two-sided t-test was used to evaluate the statistical significance between Δ*evfG* and Δ*evfG*-EvfG. *** *p* < 0.001).

The motility of parental strain PCN033, mutant Δ*evfG*,and the complementary strain Δ*evfG*-EvfG was assessed through swimming and swarming assays. The results demonstrated that the swimming and swarming capabilities of the Δ*evfG* strain were markedly diminished in comparison to the parental strain PCN033, while the Δ*evfG*-EvfG strain exhibited motility that was largely comparable to that of PCN033 (Figure 1A,C). In addition, the diameters of the bacterial swimming and swarming zones were quantitatively measured. The results demonstrated that the diameter for the mutant Δ*evfG* was notably smaller than that of parental strain PCN033, while the diameter of the complementary strain Δ*evfG*-EvfG was substantially restored relative to that of the mutant Δ*evfG* (Figure 1B,D). These results suggested that the deletion of gene *evfG* in the T6SS gene cluster had a detrimental effect on PCN033 motility.

### 3.2. Deletion of evfG Affects the Flagellum Formation in PCN033

**Figure 2:**
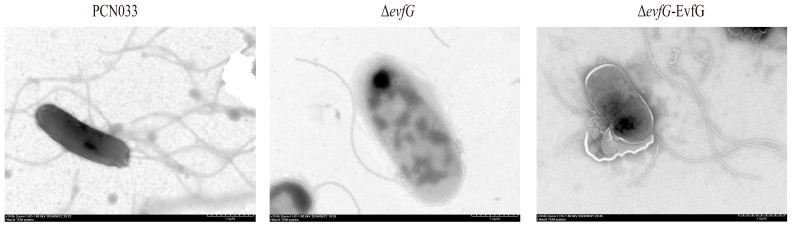
TEM observation of parental strain PCN033, mutant Δ*evfG*, and the complementary strain Δ*evfG*-EvfG. TEM was used to visualize flagella that had been negatively stained.

To explore how the deletion of *evfG* affects the motility of PCN033, we performed a comparative analysis of flagella among PCN033, Δ*evfG*, and Δ*evfG*-EvfG, which was conducted by using negative staining electron microscopy (negative-stain EM). As illustrated in Figure 2, the deletion of *evfG* resulted in an absence of flagella, whereas complementation with the *evfG* gene reestablished the flagella on the Δ*evfG* strain. These results suggest that *evfG* is essential for flagellum formation in PCN033.

### 3.3. Metabolome Profiling

**Figure 3:**
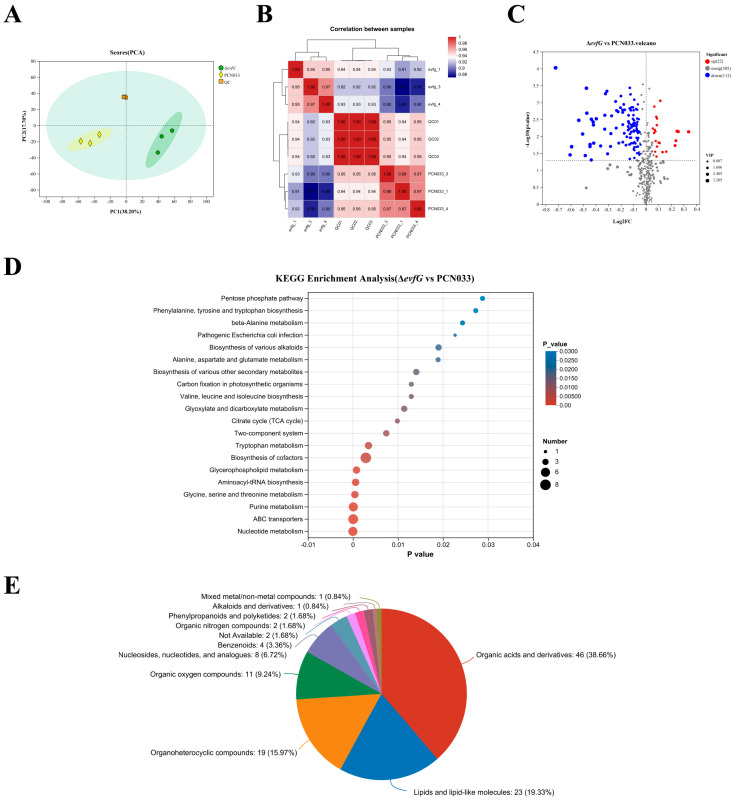
Differentially accumulated metabolites between PCN033 and mutant Δ*evfG*: (**A**) an analysis using principal component methods was performed on the metabolites found in both PCN033 and Δ*evfG*. To create a QC sample, equal amounts of the samples from PCN033 and Δ*evfG* were combined; (**B**) examination of the relationship between the *evfG* and PCN033 samples. The color gradient ranges from low (blue) to high (red), indicating the level of correlation for each sample; (**C**) volcano plots display the DAMs that were either upregulated, downregulated, or remained unchanged when comparing PCN033 and Δ*evfG*; (**D**) KEGG enrichment analysis of DAMs; and (**E**) the classification map of HMDB compounds. In this figure, based on the number of metabolites, the names of the selected HMDB hierarchies, the quantity of metabolites, and the percentage of metabolites they account for are presented in descending order.

To further investigate the impact of gene *evfG* on flagella formation in ExPEC PCN033, metabolome profiling was conducted on samples from PCN033 and Δ*evfG* using untargeted LC-MS. The metabolites revealed by LC-MS analysis are detailed in Supplementary Table S1. In total, 439 metabolites were detected, which were categorized into 11 distinct groups. Principal component analysis (PCA) performed on samples from PCN033, Δ*evfG*, and the quality control (QC) demonstrated a clear separation among these groups (Figure 3A). The concentration data of the metabolites were utilized to assess correlations between the samples, revealing that they could be distinctly differentiated (Figure 3B), thereby indicating a high level of reliability in the metabolome data obtained. Consequently, both PCA and correlation analyses suggest that significant variations exist in the metabolite profiles between the PCN033 and Δ*evfG* samples.

### 3.4. Differentially Accumulated Metabolites (DAMs) in PCN033 and ΔevfG

All metabolite data were analyzed using criteria, such as *p*-values < 0.05, |Log2 Fold Change| > 1, and a variable importance in projection (VIP) > 1, to determine the DAMs between the PCN033 and the mutant Δ*evfG*. A total of 134 DAMs were obtained, of which 22 were upregulated and 112 were downregulated (Figure 3C, Supplementary Table S2). The results of the KEGG analysis showed that nucleotide metabolism (map01232), ABC transporters (map02010), purine metabolism (map00230), aminoacyl-tRNA biosynthesis (map00970), glycerophospholipid metabolism (map00564) and glycine, serine and threonine metabolism (map00260) were significantly enriched (Figure 3D). The results of additional research indicated that DAMs within these enrichment pathways were probably engaged in the metabolism of amino acids and energy (Table 2). Furthermore, Figure 3E shows that the 134 DAMs may be divided into >6 classes. These results suggest that DAMs linked to energy and amino acid metabolism may be important in affecting PCN033 strain motility as a result of *evfG* deletion.

### 3.5. Transcriptome Profiles Between PCN033 and ΔevfG

**Figure 4:**
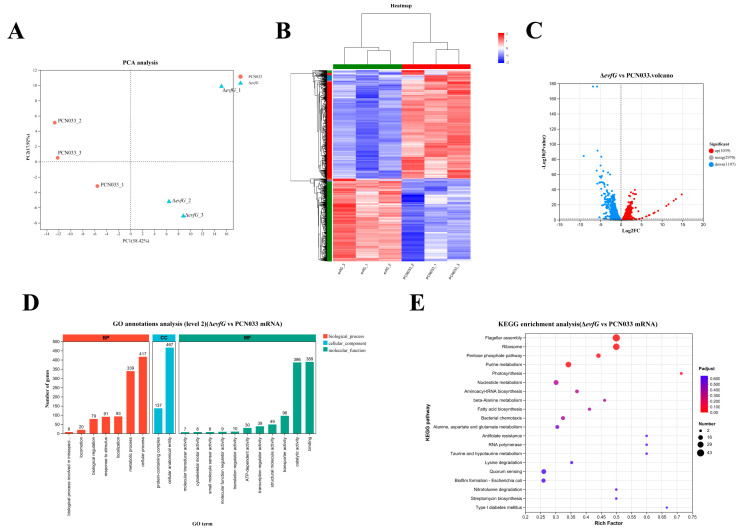
Differential phenotypes between PCN033 and mutant Δ*evfG*: (**A**) principal component analysis of genes identified from PCN033 and Δ*evfG*; (**B**) heat map of mutant Δ*evfG* and PCN033; (**C**) volcano plots; (**D**) GO enrichment; and (**E**) KEGG enrichment analysis of DEMs.

The changes in gene expression levels between Δ*evfG* and PCN033 were investigated. There were 94,346,816 (PCN033, 46,328,404; Δ*evfG*, 48,018,412) total reads generated by RNA-seq. there were 93,771,450 (PCN033, 46,012,606; Δ*evfG*, 47,758,844) clean reads obtained after removal of low-quality reads. A total of 57,094,558 (PCN033, 36,556,182; Δ*evfG*, 20,538,376) mapped reads were generated. The PCA results demonstrated that all biological replicates clustered together (Figure 4A), while the correlation analysis showed significant differences between the PCN033 and Δ*evfG* (Figure 4B).

### 3.6. DEGs Between PCN033 and ΔevfG

To predict candidate genes causing reduced motility in the mutant Δ*evfG*, DEGs were selected based on |Log2 Fold Change| > 2 or <1 between the PCN033 and Δ*evfG*. This analysis resulted in the identification of 2236 DEGs; a volcano plot was created for subsequent analyses (Figure 4C). In total, 1039 genes were upregulated and 1197 genes were downregulated. GO annotation analysis classified 2236 DEGs into three categories: molecular functions, cellular components, and biological processes (Figure 4D), among which the integral component of the cellular anatomical entity (467, 20.9%) was the largest group, followed by the cellular process (417, 18.6%) and binding (389, 17.4%); only a few DEGs (7, 0.3%) were associated with molecular transducer activity. As shown in Figure 4E, the enrichment pathway in the KEGG is flagellar assembly (map02040), ribosome (map03010), pentose phosphate pathway (map00030), and purine metabolism (map00230) (Supplementary Table S3).

### 3.7. The Correlation Analysis of DAMs and DEGs

**Figure 5:**
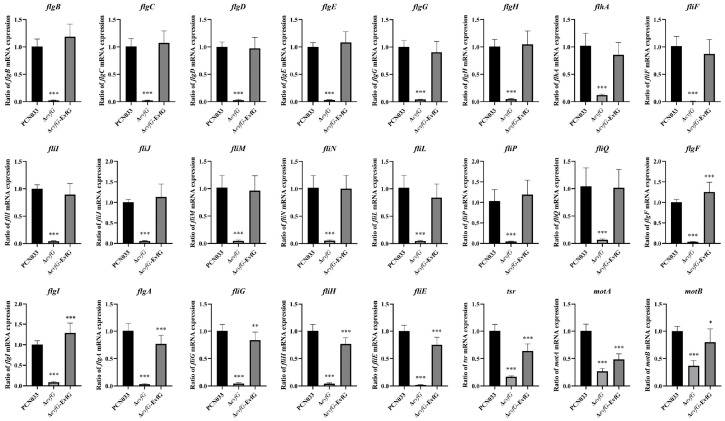
The qRT-PCR validation of flagella-associated genes of ExPEC strain PCN033, Δ*evfG*, and Δ*evfG*-EvfG. * *p* < 0.05, ** *p* < 0.01, *** *p* < 0.001.

**Figure 6:**
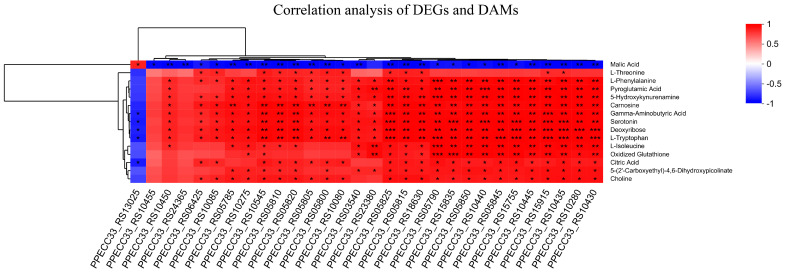
Correlation analysis of DEGs and DAMs. Rows represent metabolites, while lists represent genes. The evolutionary tree on the left represents the hierarchical clustering results of metabolites, while the evolutionary tree on the top represents the hierarchical clustering results of genes. Red represents positive correlation and blue represents negative correlation. * *p* < 0.05, ** *p* < 0.01, *** *p* < 0.001.

The most significant enrichment of DEGs in Δ*evfG* and PCN033 was observed in the pathway related to flagellar assembly (map02040). To validate the accuracy of RNA-seq data, we selected 24 DEGs associated with bacterial flagella for qRT-PCR validation (Figure 5). The results from qRT-PCR demonstrated that all these DEGs exhibited significantly lower expression levels in the mutant Δ*evfG* compared to those in the parental strain PCN033, which is consistent with our RNA-Seq findings (Supplementary Table S4). Further analysis revealed that all data collected from both metabolomic and transcriptomic profiles displayed high reliability based on the correlation analyses (Figure 6). Therefore, we propose that variations in metabolite accumulation between PCN033 and mutant Δ*evfG* are closely regulated by distinct patterns of differential gene expression.

## 4. Discussion

Bacterial motility significantly influences both the survival and the pathogenic characteristics of these microorganisms, serving a vital function throughout their lifecycle. Previous studies have found that the bacterial quorum sensing (quorum sensing, QS) system, cyclic diguanosine monophosphate (c-di-GMP) signaling system, protein glycosylation and other factors can affect bacterial motility to varying degrees [34,35,36]. This research revealed that the deletion of the gene *evfG* in the T6SS gene cluster significantly weakened the motility of porcine ExPEC. Nevertheless, the exact mechanism through which the *evfG* deletion impacts the motility of porcine ExPEC remains unclear.

### 4.1. EvfG Is Essential for the Motility of Porcine ExPEC PCN033

First, our motility tests revealed that the motility capacity of the mutant Δ*evfG* was significantly lower than that of the parental strain PCN033. In contrast, the motility capacity of the complementary strain Δ*evfG*-EvfG returned to levels comparable to those of the parental strain (Figure 1). Subsequently, by utilizing negative staining EM, a marked reduction in peripheral flagella in the mutant Δ*evfG* was observed compared to the parental strain PCN033. Notably, the number of flagella in the complementary strain Δ*evfG*-EvfG reverted to levels similar to those found in the parental strain once again (Figure 2). Through the above experiments, we have established that deletion of the *evfG* gene led to decreased motility and impaired flagella formation in ExPEC PCN033 strains. However, our research results differ from previous research findings by Lu et al. (2024) [16], which reported that the deletion of *evfG* in the T6SS gene cluster of human ExPEC RS218 strain can enhance the expression of genes related to bacterial motility and chemotaxis, indicating a negative correlation between *evfG* and bacterial motility [16]. However, the mechanism by which the gene *evfG* weakens the motility of porcine ExPEC is still unclear. Therefore, further investigation is required to elucidate how EvfG regulates both motility and flagella formation in porcine ExPEC PCN033. To gain deeper insights into the mechanisms underlying reduced motility in Δ*evfG*, we conducted transcriptome and metabolome analyses.

### 4.2. The Reduced Motility of the Mutant ΔevfG Is Related to the Energy Metabolism

Energy metabolism is the process of energy metabolism in vivo that maintains the normal growth and metabolic function of bacteria, including the citrate cycle (TCA cycle), pentose phosphate pathway and glycolysis pathway. Our combined analysis of transcriptomic and metabolomics results found that KEGG analysis of DAMs and DEGs for both Δ*evfG* and PCN033 were significantly enriched in carbohydrate metabolism and energy metabolism pathways. Furthermore, we found that the content of citric Acid and 3-phosphoglycerate of Δ*evfG* was significantly decreased; 2-hydroxybutinic acid was significantly increased in the Δ*evfG* compared to PCN033. In the TCA cycle, the condensation of acetyl CoA and oxaloacetic acid first forms citric acid. Citric acid through isomerization, oxidative decarboxylation, and hydration forms malic acid. Malic acid dehydrogenation forms oxaloacetic acid. Finally oxaloacetic acid again combines with acetyl CoA condensation to form citric acid, and completes the whole cycle process [37]. NADH and FADH_2_, generated in the citric acid cycle, enter the oxidative phosphorylation pathway to produce large amounts of ATP and provide energy to the cells [37]. However, the decrease in citrate and increased 2-hydroxysuccinate in Δ*evfG* indicate that there are obstacles in the dehydrogenation of malic acid in the TCA cycle. This causes a reduction in NADH and FADH_2_ entering the oxidative phosphorylation pathway, which in turn leads to reduced ATP generation and reduced cellular energy supply. In *E. coli*, motility is driven by flagellar rotation. Both the biogenesis of the motility system and the flagellar motor rotation consume energy in order to perform [38,39,40]. Combined with the above results, we learned that *evfG* deficiency causes citric acid cycle disorders and the cells produce less energy compared to the normal strain, while *E. coli* exercise requires energy consumption. Therefore, we reasoned that the weakened motility of the mutant Δ*evfG* may be due to its reduced energy metabolism level.

### 4.3. The Reduced Motility of the Mutant ΔevfG Was Associated with the Loss of Flagellar-Associated Genes

Both the swimming and swarming of *E. coli* are driven by flagellar rotation [25]. Bacterial motility is a complex biological property; the bacterial motility behavior and the expression of flagella-related genes are regulated by various factors. The movement of the flagellum not only helps the bacteria to better adapt to the environment, it is also closely related to its chemotaxis, adhesion, virulence, and pathogenicity characteristics [26,27]. In previous reports, the loss of flagella-related genes, such as *fliC, flgD*, and *fliN*, affected the assembly of flagella and weakened bacterial motility [32,33,41,42]. In this study, KEGG enrichment analysis of the transcriptome results of Δ*evfG* and CN033 (Figure 4E) showed that the differential genes were mainly enriched in flagellar assembly (map02040), ribosome (map03010), pentose phosphate pathway (map00030), and purine metabolism (map00230). The differential genes enriched in flagellar assembly were subsequently examined and found to be genes related to flagellar assembly and motility (see Supplementary Table S4); these genes were significantly downregulated compared with the wild strain. The findings from qRT-PCR validation aligned with the transcriptomic outcomes (Figure 5). Furthermore, by integrating the findings from the bacterial motility assay with those from the electron microscopy assay, we speculate that *evfG* of *E. coli* PCN033 plays a role in flagella synthesis. The deletion of *evfG* impairs the expression of genes related to flagella, inhibits the synthesis of flagellin, and consequently affects the motility of PCN033. However, research by Lu et al. (2024) [16], demonstrated through electron microscopy that the Δ*evfG* strain displayed a considerably greater number of flagella compared to the parental strain RS218. Both the transcriptomics and qRT-PCR results indicated that the deletion of *evfG* led to a marked increase in the expression of flagella-related genes. That is to say, the *evfG* of *E. coli* RS218 has a negative correlation with flagella synthesis [16], which is inconsistent with the findings of our study. We speculate that the molecular mechanisms by which the *evfG* of RS218 and PCN033 are involved in the regulation of flagella synthesis may be different; therefore, further research is required.

**Figure 7:**
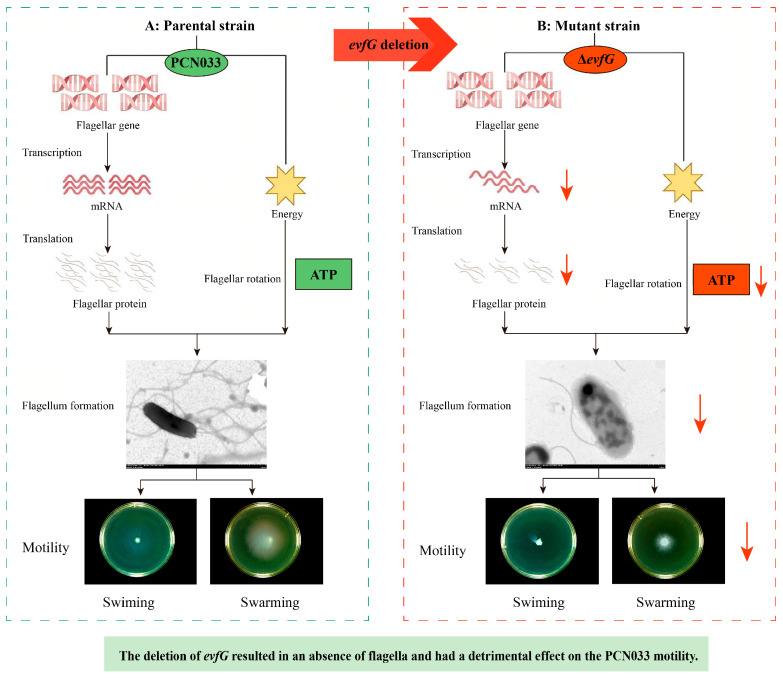
Mechanism of diminished PCN033 motility after deletion of the gene *evfG*. The red arrow denotes a significant decrease or downregulation.

In conclusion, the influencing factors of diminished motility in the mutant Δ*evfG* are shown in Figure 7. The motility ability of Δ*evfG* was weakened after the gene *evfG* deletion, which demonstrated that gene *evfG* play an important role in the motility of ExPEC. The motility of ExPEC, controlled by EvfG, occurs in a flagellum-dependent manner. In addition, the deficiency of gene *evfG* has inhibitory effects on energy production-related pathways, including the TCA cycle, the pentose phosphate pathway, the carbon fixation pathway in prokaryotes, and glyoxylate and dicarboxylate metabolism. Although the present study cannot explain the full mechanisms of EvfG action in the energy metabolism pathway and flagellar assembly, it seems closely related to the motility of ExPEC by indirectly regulating the energy metabolism pathway and flagellar assembly.

## 5. Conclusions

In the comparison between PCN033 and Δ*evfG*, 1039 upregulated and 1197 downregulated genes, 22 upregulated metabolites and 112 downregulated metabolites were found. Metabolome and transcriptomic analyses indicate that changes in energy metabolism and flagellar secretion assembly and chemotaxis are the main causes for diminished PCN033 motility after *evfG* deletion. The TCA cycle, pentose phosphate pathway, and carbon fixation pathway in prokaryotes provide energy for the flagellar movement of PCN033. The correct assembly and drive of flagellar-associated gene expression are essential for PCN033 movement. These findings offer experimental evidence and improve our comprehension of the mechanisms of pig ExPEC PCN033 motility. Our study has confirmed that *evfG* can affect the motility of PCN033 by regulating energy metabolism and the expression of flagellar genes. However, the specific mechanisms still require further in-depth investigation.

## Figures and Tables

**Table 1:** Primers used in this study.

Gene Name	Forward (5′ to 3′)	Reverese (5′ to 3′)
*flgB*	ACCTCAACGCAACACATTCC	CGCTAAGGCTCATCTGGTATTG
*flgC*	ATGGCACTGCTGAATATTTTTGATA	CACCTTTACGCCGCCTGT
*flgD*	CCACCAGTAGTAGTTCGCTCACG	GCTGACCGTGCTGATTTGTGCC
*flgG*	TACACCGAAACGCAATCCTC	TGGACACCGCTTTACTGTTG
*flgH*	GTCTACTCAGGTGGCGGATG	ACATTGGCGACAGGTTAAGGA
*flgE*	CTCAAGCGGTTAGCGGATTA	GTGGTCGTGCCATCGGTA
*flhA*	CGCCTCATCTTCACCAATCA	TTGTTCTCGATGGTATGCCG
*fliF*	TCGCACCATTCGTCATACCA	CATCTGTTCGTTGCTGAGAGG
*fliI*	CCCAATCAATGCCCTGCTTAC	CACCAATCAAACCCACGACAA
*fliJ*	GCGATATGAGTGCCGGGATG	GCTGTTCAGGGCAATGTCAA
*fliM*	GCCGACCAACCTGAACCTTA	CCACTTTAGTCGGGAAGCGT
*fliN*	CCGCACACGAATGACCATC	TATCAGCGACTACGACAACTTC
*fliL*	TGGCATTCGCATCAGGTTG	CATCTTTCAGTCGCAGGGTTAT
*fliP*	GCGCCACCTAACCAGGTATT	AACCCTAAATCCGCCTCACG
*fliQ*	CGCCACACAGATTAACGAAATG	CAGCAACAGATTGAGCATCCA
*flgF*	CACAATCTCGGTGCTCAATCC	CGACATCACACGCAAGGTT
*flgI*	CTGGTTCGGTGGTGATGAATC	GTATCTGGCTGGCTGACATTG
*flgA*	CTGCGGTAACGACAAACGATA	CAGGCTAACGGCATCAACAA
*fliG*	TGCTCGACGGTCAGAATCTC	GCGGTAATAACGGCTTCTTCC
*fliH*	GCTTGATAGTGTGATTGCTTCG	CGCTGAATAACGGTTCTTGCT
*fliE*	CCGCACCCGACCATTAGTT	AGCTTATTACGCACCTGAATCC
*tsr*	CAGCGATAACAATGCCTCCTAC	TCATTAGAGCCATCCACCTCAA
*motA*	ATAGAGCGTTTTGCGACCGA	CGATGGTGGGGACTTTCCTC
*motB*	GCTGATTCAGATTGCGGAGTA	ATGTTCGGCTGCTTATTCACTT
*16sRNA*	GAATGCCACGGTGAATAC	GGTTACCTTGTTACGACTTC

**Table 2:** The list of nine DAMs detected in the Δ*evfG* compared to PCN033.

Pathway	Metabolite	Formula	Peak Area		VIP	FC	*p*_Value	Type
			Δ*evfG*	PCN033				
Citrate cycle (TCA cycle)	Citric Acid	C_6_H_8_O_7_	5.05 ± 0.168	5.479 ± 0.076	1.2968	0.922	0.02568	down
	Malic Acid	C_4_H_6_O_5_	5.678 ± 0.036	5.411 ± 0.081	1.4736	1.049	0.0006089	up
Glyoxylate and dicarboxylate metabolism	Citric Acid	C_6_H_8_O_7_	5.05 ± 0.168	5.479 ± 0.076	1.2968	0.922	0.02568	down
	3-Phosphoglycerate	C_3_H_7_O_7_P	3.034 ± 0.201	4.617 ± 0.849	2.3762	0.657	0.07599	down
	Malic Acid	C_4_H_6_O_5_	5.678 ± 0.036	5.411 ± 0.081	1.4736	1.049	0.0006089	up
Pentose phosphate pathway	Deoxyribose	C_5_H_10_O_4_	5.58 ± 0.04	5.957 ± 0.06	1.1794	0.937	0.03162	down
	3-Phosphoglycerate	C_3_H_7_O_7_P	3.034 ± 0.201	4.617 ± 0.849	2.3762	0.657	0.07599	down
Glycine, serine and threonine metabolism	Choline	C_5_H_14_NO+	6.157 ± 0.123	6.492 ± 0.04	1.1949	0.948	0.01386	down
	L-Tryptophan	C_11_H_12_N_2_O_2_	4.294 ± 0.061	4.935 ± 0.098	1.7677	0.87	0.002812	down
	3-Phosphoglycerate	C_3_H_7_O_7_P	3.034 ± 0.201	4.617 ± 0.849	2.3762	0.657	0.07599	down
Aminoacyl-tRNA biosynthesis	L-Tryptophan	C_11_H_12_N_2_O_2_	4.294 ± 0.061	4.935 ± 0.098	1.7677	0.87	0.002812	down
	L-Phenylalanine	C_9_H_11_NO_2_	4.114 ± 0.125	4.496 ± 0.078	1.2485	0.915	0.0234	down
Tryptophan metabolism	Serotonin	C_10_H_12_N_2_O	4.104 ± 0.042	4.729 ± 0.131	1.702	0.868	0.005014	down
	L-Tryptophan	C_11_H_12_N_2_O_2_	4.294 ± 0.061	4.935 ± 0.098	1.7677	0.87	0.002812	down
Alanine, aspartate and glutamate metabolism	Gamma-Aminobutyric Acid	C_4_H_9_NO_2_	5.967 ± 0.067	6.419 ± 0.073	1.4728	0.93	0.002632	down
	Citric Acid	C_6_H_8_O_7_	5.05 ± 0.168	5.479 ± 0.076	1.2968	0.922	0.02568	down

**Note:** Pathway: Description of the pathway enriched by KEGG enrichment analysis; Metabolite: Metabolites enriched under the corresponding pathway; Formula: Chemical formula of the metabolite; Peak area: Peak area of the metabolite (mean ± standard deviation); VIP: Variable important in projection; FC: Fold Change; *p*_value: Derived from t-test statistical analysis; Type: Change trend of metabolites.

## Data Availability

The data used and/or analyzed during the current study are available from the corresponding author on reasonable request.

## Supplementary Materials

The following supporting information can be downloaded at: https://www.mdpi.com/article/10.3390/biology14070822/s1.
