# Supplementary material for: The Regulatory Role of EvfG Through Coordinated Control of Flagellar Biosynthesis and Energy Metabolism in Porcine Extraintestinal Pathogenic Escherichia coli (ExPEC)
Source: Biology (Basel). 2025 Jul 7;14(7):822. doi: 10.3390/biology14070822 (PMC12292395; doi:10.3390/biology14070822)
Supplement: Supplementary file 1 [file biology-14-00822-s001.zip › Supplementary Table S4.pdf]

Table S4. The differentially expressed genes of 7 GO enrichment pathways detected in the Δ*evfG* compared to PCN033

GO Function	Gene ID	Ko name	Gene description	FC (Δ*evfG*/ PCN033)	*P*_value
**Bacterial-type flagellum organization**					
	PPECC33_RS01420	flgM	flagellar biosynthesis anti-sigma factor FlgM	0.273	0.000212209
	PPECC33_RS10565	fliR	flagellar biosynthetic protein FliR	0.083	4.69339E-18
	PPECC33_RS10450	fliS	flagellar export chaperone FliS	0.429	1.26443E-08
	PPECC33_RS05785	flgN	flagella biosynthesis chaperone FlgN	0.363	1.53295E-12
	PPECC33_RS05795	flgA	flagellar basal body P-ring formation chaperone FlgA	0.036	1.58971E-31
	PPECC33_RS10225	flhA	flagellar biosynthesis protein FlhA	0.133	3.84629E-25
	PPECC33_RS10440	PPECC33_RS10440	FliC/FljB family flagellin	0.283	5.80724E-21
	PPECC33_RS01495	lafA	lateral flagellin LafA	0.1	6.42579E-35
	PPECC33_RS01505	fliD	flagellar filament capping protein FliD	0.179	1.13126E-05
**Flagellum-dependent cell motility**					
	PPECC33_RS10505	fliF	flagellar basal-body MS-ring/collar protein FliF	0.017	1.22028E-46
	PPECC33_RS10510	fliG	flagellar motor switch protein FliG	0.028	3.46146E-48
	PPECC33_RS10525	fliJ	flagellar export protein FliJ	0.034	6.87667E-40
	PPECC33_RS05835	flgI	flagellar basal body P-ring protein FlgI	0.088	6.39132E-37
	PPECC33_RS05830	flgH	flagellar basal body L-ring protein FlgH	0.06	1.08363E-42
	PPECC33_RS18010	rpoA	DNA-directed RNA polymerase subunit alpha	0.247	7.67719E-09
	PPECC33_RS21995	rpoB	DNA-directed RNA polymerase subunit beta	0.264	1.07085E-12
	PPECC33_RS10280	motA	flagellar motor stator protein MotA	0.303	1.97387E-32
	PPECC33_RS10500	fliE	flagellar hook-basal body complex protein FliE	0.052	2.09921E-08
	PPECC33_RS10515	fliH	flagellar assembly protein FliH	0.033	1.84204E-54
	PPECC33_RS06330	ycgR	flagellar brake protein	0.415	4.56059E-07
	PPECC33_RS10535	fliL	flagellar basal body-associated protein FliL	0.06	1.80126E-28
	PPECC33_RS10275	motB	flagellar motor protein MotB	0.388	9.51938E-13

**Chemotaxis**				
PPECC33_RS24205	Tsr	methyl-accepting chemotaxis protein	0.105	4.16086E-61
PPECC33_RS10260	Tar	methyl-accepting chemotaxis protein II	0.429	1.78965E-13
PPECC33_RS10265	cheW	chemotaxis protein CheW	0.424	6.28466E-14
PPECC33_RS10270	cheA	chemotaxis protein CheA	0.312	2.06591E-20
PPECC33_RS05840	flgJ	flagellar assembly peptidoglycan hydrolase FlgJ	0.086	4.46244E-38
PPECC33_RS16920	Aer	aerotaxis sensor receptor Aer	0.222	1.32377E-20
PPECC33_RS10540	fliM	flagellar motor switch protein FliM	0.045	5.1021E-48
PPECC33_RS10545	fliN	flagellar motor switch protein FliN	0.056	1.54974E-45
PPECC33_RS11580	mglB	galactose/glucose ABC transporter substrate-binding protein MglB	0.375	0.000370357
PPECC33_RS07750	Trg	methyl-accepting chemotaxis protein Trg	0.206	4.58105E-24
**Bacterial-type flagellum hook**				
PPECC33_RS10530	fliK	flagellar hook-length control protein FliK	0.036	1.4921E-48
PPECC33_RS10445	fliD	flagellar filament capping protein FliD	0.3	2.80055E-28
PPECC33_RS05850	flgL	flagellar hook-associated protein FlgL	0.335	3.03831E-20
PPECC33_RS05815	flgE	flagellar hook protein FlgE	0.034	1.23924E-81
PPECC33_RS05845	flgK	flagellar hook-associated protein FlgK	0.276	7.04884E-18
PPECC33_RS05810	flgD	flagellar hook assembly protein FlgD	0.03	3.66581E-70
**Bacterial-type flagellum-dependent swarming motility**				
PPECC33_RS05825	flgG	flagellar basal-body rod protein FlgG	0.048	6.14995E-63
PPECC33_RS05800	flgB	flagellar basal body rod protein FlgB	0.015	2.12707E-63
PPECC33_RS05805	flgC	flagellar basal body rod protein FlgC	0.026	6.33333E-56
PPECC33_RS05820	flgF	flagellar basal-body rod protein FlgF	0.037	1.45829E-56
**Negative regulation of bacterial-type flagellum assembly**				
PPECC33_RS04425	ybjN	YbjN domain-containing protein	0.449	5.33483E-09
PPECC33_RS10455	fliT	flagella biosynthesis regulatory protein FliT	0.442	1.68584E-05

**Bacterial-type flagellum secretion apparatus**				
PPECC33_RS10550	fliO	flagellar biosynthetic protein FliO	0.081	5.6779E-16
PPECC33_RS10555	fliP	flagellar type III secretion system pore protein FliP	0.053	8.74385E-34
PPECC33_RS10560	fliQ	flagellar biosynthesis protein FliQ	0.048	3.54131E-19
PPECC33_RS10520	fliI	flagellar protein export ATPase FliI	0.024	6.64429E-48
